# Analyzing Developing Brain-On-Chip Cultures with the CALIMA Calcium Imaging Tool

**DOI:** 10.3390/mi12040412

**Published:** 2021-04-08

**Authors:** Elles A. L. Raaijmakers, Nikki Wanders, Rob M. C. Mestrom, Regina Luttge

**Affiliations:** 1 Electromagnetics Group, Department of Electrical Engineering, Eindhoven University of Technology, 5600 MB Eindhoven, The Netherlands; e.a.l.raaijmakers@tue.nl (E.A.L.R.); R.M.C.Mestrom@tue.nl (R.M.C.M.); 2 Neuro-Nanoscale Engineering Group, Department of Mechanical Engineering and Institute of Complex Molecular Systems (ICMS), Eindhoven University of Technology, 5600 MB Eindhoven, The Netherlands; n.wanders@tue.nl

**Keywords:** brain-on-chip culture, calcium fluorescence imaging, software tool, neuronal network

## Abstract

Brain-on-chip (BoC) models are tools for reproducing the native microenvironment of neurons, in order to study the (patho)physiology and drug-response of the brain. Recent developments in BoC techniques focus on steering neurons in their activity via microfabrication and via computer-steered feedback mechanisms. These cultures are often studied through calcium imaging (CI), a method for visualizing the cellular activity through infusing cells with a fluorescent dye. CAlciumImagingAnalyser 2.0 (CALIMA 2.0) is an updated version of a software tool that detects and analyzes fluorescent signals and correlates cellular activity to identify possible network formation in BoC cultures. Using three previous published data sets, it was demonstrated that CALIMA 2.0 can analyze large data sets of CI-data and interpret cell activity to help study the activity and maturity of BoC cultures. Last, an analysis of the processing speed shows that CALIMA 2.0 is sufficiently fast to process data sets with an acquisition rate up to 5 Hz in real-time on a medium-performance computer.

## 1. Introduction

Developing new types of medication for an organ as complex as the brain remains a challenge until now. In the past, brain diseases were studied mainly in ex-vivo slices of animal brains and in cell cultures. From these options, ex-vivo slices are a more accurate representation of a brain as these contain remnants of a functional network, including extracellular matrix (ECM) and a variety of cell types [1]. Moreover, brain slices are valuable, not only because of their cellular and matrix components, but because they preserve, even though in part, the original circuitry of the native tissue, as opposed to cell cultures, where this architecture is lost. However, not all properties of animal brains are translatable to the human brain and the use of animals for research and drug development yields ethical issues on top of the scientific challenges.

Hence, more elaborate brain models are being developed based on cultures of human cells rather than ex-vivo brain slices. Brain-on-chip (BoC) models intend to mimic the physiological microenvironment of the neurons such that the cell cultures can operate under “natural” conditions. A well-designed BoC system can exhibit electrical activity and develop communication networks [1,2]. When such models encompass diseases and disorders of the brain, it is believed that the knowledge of the connectivity within the brain areas is required to better understand the effects of pharmacological drugs on certain brain areas [3].

Another recent trend in the development of BoC cultures is its influence on activities in the cultures by integrated nano-and microfabricated physical features that, either directly affect cell differentiation processes as ECM biomimicking factors, e.g., nanogrooves [4], and by time-responsive control mechanisms to evoke a response of the cultured network [5]. Recent advances in calcium signal analysis software have brought fast processing algorithms that enable real-time, feedback-based (closed-loop) control of cellular activity [6,7]. Such experiments require a fast processing of the data acquired from such cultures.

The main optical imaging technique allowing for dynamic analysis of neuronal activity in multiple cells is fluorescent calcium imaging (CI), which is a method to measure cell activity in multiple neurons at the same time [8]. Cells are treated with a fluorescent dye, and the fluorescent properties of the dye change when the amount of intracellular calcium is altered, such as the case during neuronal signalling [8]. Comparing neuronal signalling between different cells can correlate to these neurons being connected in a network, which is essential for revealing information of maturity in the neural cell networks.

There are many semi-automated CI-analysis tools readily available, but not all allow for correlation analyses of cellular activity, which is required to study network development [9,10,11], or require supporting software such as Matlab [12]. Additionally, the program should be open source to ensure accessibility, data handling transparency, and continuous improvement of the software, in line with the expected high potential for new discoveries. For example, BoC platform technology can be used to explore novel therapeutic strategies for neurodegenerative diseases [13], to investigate drug delivery across the blood-brain barrier [14] or to study the complexity of the brain [15]. Finally, a CI-tool should be fast enough to allow the aforementioned closed-loop experiments, and sufficiently accurate with respect to the unmet need for these on-chip brain models in the pharmaceutical industry.

The previously developed open-source CALciumIMagingAnalyser (CALIMA) 1.0 [16] enables the user to detect cells, process calcium spike activity and provides an overview of the communication between neurons within limited computation time. It demonstrated the ability to detect and process calcium spike activity for two examples where neural activity was evaluated using CI in BoC technology utilizing three-dimensional (3D)-microsieve-assisted scaffolding by Moonen et al. [17], and applying nanogrooved substrates for ECM mimicry by Bastiaens et al. [4]. However, while CALIMA 1.0 is able to detect calcium peaks, its algorithms translation from raw fluorescence traces to the changes in cellular free calcium concentrations is lacking. Cellular calcium plays a role in communications between cells, as well as in processes regarding cell health, such as apoptosis and differentiation [8]. Therefore, a translation of fluorescent traces to cellular calcium concentrations is required to further develop culture-based techniques such as BoC.

In this paper, we present CALIMA 2.0, a user-friendly open-source CI-tool available at [18], which is able to identify individual cell activity in a network of cells, and meeting the requirements set above. Modifications in the source code of CALIMA 1.0 allow for a more detailed analysis of the fluorescent intensity fluctuations delineated by the applied algorithms, along with improvements in processing speed. This provides the next step towards the interpretation of the physiological meaning of these signals. Here, the improvements in the algorithms are detailed, initial estimates regarding the parameters used by CALIMA 2.0 are given and the data processing is demonstrated in three data sets. Additionally, the processing speed of CALIMA 2.0 is investigated.

## 2. Materials and Methods

### 2.1. Processing Workflow

**Figure 1:**
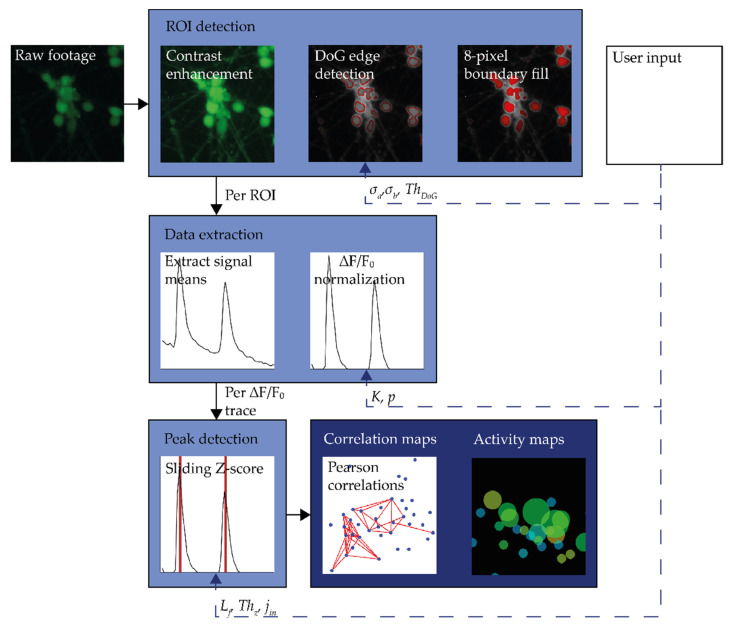
The workflow of CALIMA 2.0 with the processing steps indicated by light blue blocks and the outputs by dark blue blocks. Furthermore, the user-set input parameters are indicated for each processing step.

CALIMA 2.0 uses the framework and data loading algorithms of CALIMA 1.0 [16], but data processing strategies have been adapted to improve the processing speed and to make parameter selection more intuitive. The schematic workflow of CALIMA 2.0 is depicted in Figure 1, and will be further detailed in the following sections.

### 2.2. Processing Changes Compared to CALIMA 1.0

#### 2.2.1. ROI Detection

After loading a data set, the regions of interest (ROI) are detected, see the top part in Figure 1. The footage is first averaged per pixel and contrast-stretched, resulting in an image normalized to a scale from 0 to 1. Next, a difference of Gaussians (DoG) filter is used to detect the ROIs, which enhances the features of the image at edges to discover locations with sharp dark-to-bright transitions [19]. CALIMA 1.0 used an additional blurring filter on top of a DoG filter to reduce the influence of neurites on the ROI detection [16]. In CALIMA 2.0, this step has been combined with the DoG filters to save processing time and to reduce the number of parameters to be set by the user.

#### 2.2.2. Signal Extraction

Per ROI, the raw fluorescence signals are extracted from the raw footage by averaging the values of all pixels in the ROI per frame, see Figure 1. The raw fluorescence signals are then corrected for the background fluorescence and baseline fluorescence of the ROI through a sliding window algorithm similar to the one used in [12]. This approach is different from the strategy used by CALIMA 1.0, which combined the signal normalization and calcium peak detection steps to save on processing time [16]. However, the normalization to the background and baseline fluorescence, enables the extraction of fluorescence traces, which are directly related to the cell’s free calcium concentration [20]. The fluorescence intensity $F_{0}$ of a cell in rest is determined, and the fluorescence data $F_{raw}$ of a cell $F_{raw}$ is compared to it through$\;\Delta F=F_{raw}-F_{0}$. This ΔF serves as a measure of the cellular fluorescence activity of the cell compared to its activity in rest. As $F_{0}$ varies greatly between cells, the absolute fluorescence difference ΔF is normalized to $F_{0}$ to obtain a relative measure of the fluorescence activity of the cell. Changes in these $\Delta F/F_{0}$ traces can be related to the internal calcium concentrations of the cells. These concentration differences can be linked to a plethora of biochemical processes [8], and are, thus, of interest.

#### 2.2.3. Peak Detection

The calcium spikes are detected from the $\Delta F/F_{0}$ traces through a robust sliding Z-score algorithm [21]. This algorithm tracks the signal mean and standard deviation when the ROIs are in rest and uses this information to detect signal peaks. An extended version of this algorithm was used in CALIMA 1.0 for the combined signal normalization and peak detection [16]. In CALIMA 2.0, the number of parameters that has to be set for peak detection has been reduced to three, simplifying this strategy. Now, the user only has to set the size of the window from which the local signal mean and standard deviation are determined, a smoothing factor which reduces the influence of peaks on the local signal mean and standard deviation, and the peak detection threshold.

### 2.3. Algorithms and Parameter Space

#### 2.3.1. ROI Detection

The ROI boundaries $ROI_{bnd}$ are found from the pixels having intensities above a certain threshold $Th_{DoG}$ in the image after a DoG operation D with blurring parameters $\sigma_{a}$ and $\sigma_{b}$ on the averaged, contrast-stretched image $I_{avg}$ from the following set of equations:

(1) $$ROI_{bnd}=D\left(\sigma_{a},\;\sigma_{b}\right)>Th_{DoG}$$

(2) $$D\left(\sigma_{a},\;\sigma_{b}\right)=\;I_{avg}\;*\;G\left(\sigma_{a}\right)-\;I_{avg}\;*\;G\left(\sigma_{b}\right).$$

Here, G represents the Gaussian blur filter as given by,

(3) $$G\left(\sigma \right)=\frac{1}{\left(2\pi \sigma^{2}\right)\;}\exp\left(\frac{-x^{2}\;+\;y^{2}}{2\sigma^{2}}\right)$$

with a Gaussian kernel of width σ, and spatial coordinates (pixel positions) x and y. Boundary pixels were padded to enable the detection of ROIs at the sides and corners of the footage. Next, the areas enclosed by $ROI_{bnd}$ are filled using an 8-pixel boundary fill algorithm.

The optimal settings for the ROI detection depend on the dataset. A sensible initial estimate for the three parameters $\sigma_{a}$, $\sigma_{b}$ and $Th_{DoG}$ can be determined as follows. The DoG filter acts as a spatial band pass filter for the brightness gradients between cut-off frequencies of $1/2\pi \sigma_{b}$ and $1/2\pi \sigma_{a}$ [19]. Based on the examples obtained from [5,22], the per-pixel maximum brightness rate of change of a contrast stretched calcium fluorescence image is typically in the range of 0.1 to 0.33. This implies that both $\sigma_{a}$ and $\sigma_{b}$ should be between 3–10 pixels. Furthermore, setting $\sigma_{b}$ to 1.6 times $\sigma_{a}$ makes the DoG filter approach the behavior of the Laplacian of Gaussian edge detection operator [19], [23]. The optimal value of $Th_{DoG}$ depends on the scale factor between $\sigma_{a}$ and $\sigma_{b}$ [19]. Starting with $Th_{DoG}=0.002\left(\sigma_{b}/\sigma_{a}\right)$ appears to be a suitable initial guess. The number of ROIs found and the area of the ROIs discovered can both be increased by lowering $Th_{DoG}$.

#### 2.3.2. Signal Extraction

To find the $\Delta F/F_{0}$ traces, the per-ROI averaged signal traces need to be corrected for the background fluorescence $F_{min}$ and the ROI-dependent resting fluorescence $F_{0}$ [20], which is done with a sliding window like described in:

(4) $$\Delta F\;/F_{0}\left[n\right]=(F_{raw}\left[n\right]-F_{0}\left[n\right])/F_{0}\left[n\right]$$

(5) $$F_{0}\left[n\right]=F_{low}\left[n\right]-F_{min}$$

(6) $$F_{low}\left[n\right]=mean\left(lowest\;q\;\%\;of\;values\;of\;(F_{raw}\left[n-K:n\right]\right)).$$

Here, $F_{raw}$ is the per-time frame averaged signal trace of an ROI in frame n, $F_{low}$ the magnitude of the fluorescence signal in absence of a calcium spike, K the user-set window size and *q* an user-set parameter determining the percentage of lowest values of $F_{raw}$ used to find $F_{low}$.

The background fluorescence $F_{min}$ is automatically estimated by averaging the 1% lowest-valued pixels of the first frame of the footage. Furthermore, it is advisable to choose window length K, such that it can contain at least one full calcium spike. The rise times and decay constants of the calcium spikes depend among others on the fluorescent dye [24] and last milliseconds to min [24,25], placing window size K in the range of tens (footage acquisition rate ~1 Hz [4,5,25,26]) to hundreds of frames (acquisition rate ~10 Hz [27]). Parameter q should be chosen sufficiently high such that multiple samples are used to estimate $F_{low}$*,* but well below 50 to avoid basing $F_{0}$ on trace sections that contain calcium spikes. If K is much larger than the duration of one calcium spike, q should typically be 10–20.

#### 2.3.3. Peak Detection

The peaks in the $\Delta F/F_{0}$ traces are found with a robust sliding Z-score algorithm [21], a variation of the often-used Z-score algorithm [28] that determines the signal mean and standard deviation locally to detect signal peaks. The robust Z-score can be applied to the $\Delta F/F_{0}$ traces through the following set of equations:

(7) $$P_{f}\left[n\right]=\{\begin{array}{c} 1\;if\;Z_{f}\left[n\right]>Th_{Z}\\ 0\;otherwise \end{array},$$

(8) $$Z_{f}\left[n\right]=\left({\frac{\Delta F/F_{0}\left[n\right]-\;{µ}_{f}\;\left[n\;-\;1\right]}{SD_{f}\left[n-1\right]}}_{\;}\right),$$

(9) $${µ}_{f}\left[n\right]=\frac{1}{L_{f}}\;\overset{n-1}{\underset{i=n-L_{f}-1\;}{\sum }}F_{buff}\;\left[i\right]$$

(10) $$SD_{f}\left[n\right]\;=\;\sqrt{\frac{1}{L_{f}-1}\;\overset{n-1}{\underset{i=n-L_{f}-1\;}{\sum }}{\left(F_{buff}\;\left[i\right]-\;{µ}_{f}\;\left[n\right]\right)}^{2}\;}$$

(11) $$F_{buff}\left[n\right]=\{\begin{array}{c} j_{in}\Delta F/F_{0}\left[n\right]\;+\;\left(1\;-\;j_{in}\right)F_{buff}\;\left[n\;-\;1\right]\;if\;\;P_{f}\;\left[n\right]\;=\;1\\ \Delta F/F_{0}\left[n\right]\;\;otherwise \end{array}$$

Here, the binary array $P_{f}$ contains a 1 if a peak is detected in frame n and a 0 if there is not. The array $Z_{f}$ contains the signal’s Z-score, $Th_{Z}$ is the user-set threshold for peak detection ${µ}_{f}$ and $SD_{f}$ represent the trace mean and standard deviation, $L_{f}$ is the user-set window length used to estimate ${µ}_{f}$ and $SD_{f}$, $F_{buff}$ is a low-pass filtered version of the $\Delta F/F_{0}$ trace, and $j_{in}$ is the user-set smoothing factor of the filter. Low-pass filtering the traces in the presence of a peak decreases the influence peaks have on the estimates of the signal mean and standard deviation, which leads to more robust peak detections. Note that $SD_{f}$ is set to be minimally $1/\left(10Th_{Z}\right)$ to avoid accidental divisions by 0, see Equation (8). The threshold for peak detection $Th_{Z}$ represents by what amount the signal must be larger than the standard deviation before it is classified as a peak. Depending on the signal to noise ratio of the traces, a value of at least 3.0 should suffice. Ideally but not necessarily, the window length $L_{f}$ is chosen longer than the duration of one calcium spike. The smoothing factor $j_{in}$ should be set to a small value (0.1–0.2), but preferably higher than 0 to ensure that calcium spikes do not contaminate the sliding window estimates.

#### 2.3.4. Parameter Space Summary

The start estimates for the parameters used by CALIMA 2.0 are summarized in Table 1.

### 2.4. Data Acquisition and Management

To evaluate the data processing of CALIMA 2.0, a data set for visualizing calcium influx and testing the computational processing speed was retrieved from an online source [22] (*dataset 1*). *Dataset 1* contains spontaneous activity of cultured primary rat cortical neurons transduced with AAV2-GCaMP6 dye. The 696 by 520 pixel 16-bit TIFF-format dataset was acquired at 10 Hz.

Additionally, published data from human induced pluripotent stem cell-derived neuronal cells (hiPSCNs) on nanogrooved polydimethylsiloxane (NG-PDMS) was used to demonstrate the functionality of CALIMA 2.0 in a BoC environment [4] (*dataset 2*). In brief, nanogrooved substrates, obtained via lithography with a 1000 nm pattern periodicity and 230 nm ridge [5], were used to study guidance of neuronal outgrowths in hiPSCNs. Cells were loaded with (Fluo-4 Calcium Imaging Kit, Thermo Fisher, Walham, MA, USA [29]) and a TIFF-format dataset with 1280 by 960 pixels was recorded during a 10-min time-lapse with a 0.1 Hz acquisition rate after the culture had been developing for 13 days in vitro (DIV). CALIMA 2.0’s block peak detection was used, which measures per ROI all the frames reaching higher than the peak threshold rather than just the moments of the peaks. This was done to measure the duration of calcium level elevation. Newly obtained data were exported as open file formats (.csv) for further analysis.

Lastly, to demonstrate the spatiotemporal analysis properties of CALIMA 2.0, a data set was obtained from Xie et al. [5]. This BoC data set is based on a PMDS actuator chip capable of mechano-stimuli to study network maturation. An applied pressure of 200 mbar for 10 s throughout the calcium recording resulted in a detectable deformation within the chip. Primary rat cortical neurons were loaded with Fluo-4 AM dye. *Dataset 3* has a bit depth of 16, is 512 by 512 pixels wide, and the acquisition rate was 0.254 Hz during an experiment lasting 46 s (183 frames). For the spatio-temporal analysis, only a few specific ROIs are of interest for comparing the results to the analyses done by the original authors. Hence, a mask containing 38 ROIs was applied to this data and only the frames 75 to 183 were loaded (just before the stimulus onset until the end of the experiment).

## 3. Results

### 3.1. Physiological Behavior

**Figure 2:**
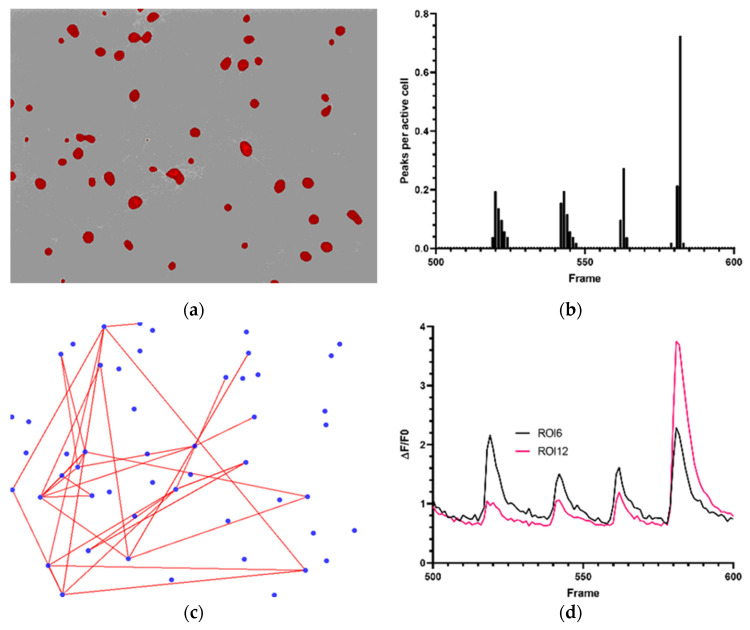
Neuronal activity of primary rat cortical cells. (**a**) 51 ROIs detected by CALIMA 2.0. (**b**) Average peaks per active cell in primary rat cortical cells between frame 500 and 600. (**c**) Proposed correlations between active primary rat cortical cells according to CALIMA 2.0 software (Pearson correlation factor of ≥0.7). (**d**) Calcium fluorescence trace of ROI6 and ROI12 between frames 500 and 600.

Using the parameters as mentioned in Table 2, a total of 51 cells were detected in *dataset 1* (Figure 2a). A cumulative total of 0 to 40 calcium spikes was measured over time, with a maximum average of 0.73 peaks per active cell per frame (Figure 2b). Proposed correlated cell activity can be seen in Figure 2c (Pearson correlation factor of ≥0.7). Multiple cells with highly correlated activity can be determined from this plot. This could serve as an indication towards communication in a network between the separate cells. Figure 2d represents two correlated ROIs. The graph demonstrates that activity is concentrated around certain time instances, underlining the correlations in activity demonstrated by Figure 2c. Furthermore, the duration and shapes of the $\Delta F/F_{0}$ traces extracted by CALIMA 2.0 are in line with examples from literature [27].

**Figure 3:**
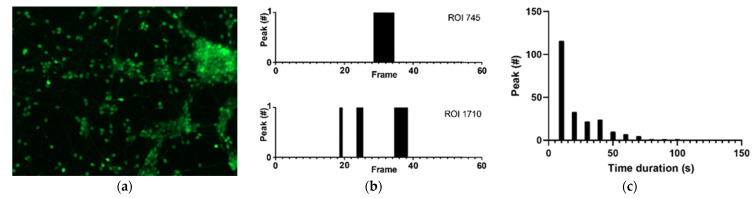
Neuronal activity of hiPSCNs on NG-PDMS. (**a**) hiPSCNs loaded with green-fluorescent Fluo-4 AM. Magnification x20. (**b**) Active calcium spikes (calcium level is above peak threshold) displayed by black columns in two ROIs. (**c**) Calcium spike durations of all ROIs displaying calcium spikes.

*Dataset 2* consisted of hiPSCNs on NG-PDMS detected with the parameters mentioned in Table 3. The images shown in Figure 3 are cells at 13 DIV for one representative sample. 3108 ROIs were detected. A cell cluster can be observed on the top right in (a), along with dispersed cell nuclei and outgrowths; (b) demonstrates peaks lasting multiple frames seen in multiple ROIs in *dataset 2*; (c) shows that peaks last up to 100 s (average 21.80 s), indicating that calcium transients vary in time.

**Figure 4:**
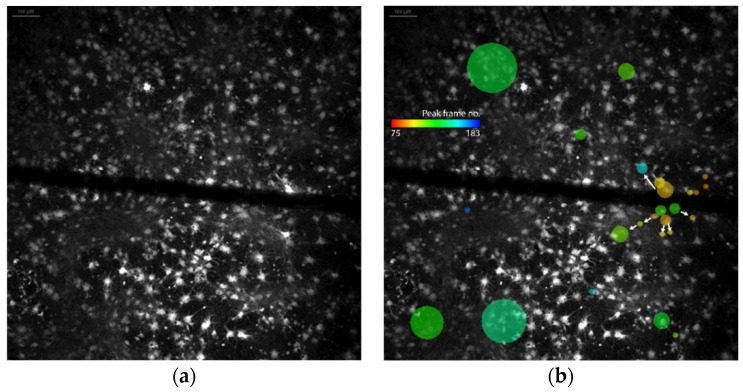
Stimulus-induced activity in primary cortical rat cells. (**a**) Wistar rat cortical cells loaded with green-fluorescent Fluo-4 AM. (**b**). Spatio-temporal map of the activity in the culture with the color of a dot representing the mean of the Figure 1. The arrows indicate the apparent directions in which the activity travels. Note that the green ROIs where the cascade starts are reactivated, and thus, show some activity later in the experiment as well as early.

*Dataset 3* consisted of rat cortical cells on NG-PDMS detected with the parameters mentioned in Table 4. Neuronal activity of hiPSCNs on NG-PDMS. (a) hiPSCNs loaded with green-fluorescent Fluo-4 AM. Magnification x20. (b) Active calcium spikes (calcium level is above peak threshold) displayed by black columns in two ROIs. (**c**) Calcium spike durations of all ROIs displaying calcium spikes. A mask containing 38 ROIs was applied. The cells and the microchannel through which a stimulus was induced can be observed in Figure 4a. A spatio-temporal map of the cellular activity can be seen in Figure 4b. Here, it can be seen through the different colored bubbles that the activity in the culture appears to spread from ROIs located in the middle right of the image near the channel. This pattern of spatio-temporal activity corresponds with the findings described in [5].

### 3.2. Processing Speed

The algorithms have been implemented with processing speed in mind. Firstly, CALIMA 2.0 detects the number of logical cores used by the computer, and the data loading, ROI detection, signal extraction and calcium spike detection algorithms have been multi-threaded. To that end, a separable version of the DoG filter has been implemented [30], which splits the DoG operation in four vector convolutions rather than the original two computationally more expensive matrix convolutions. To save some additional computation time, the length of these vectors was limited to six times $\sigma_{b}$ as a Gaussian function decreases to nearly zero at more than three standard deviations distance from its origin. Furthermore, the signal extraction algorithm requires the lowest values of a set to be found rapidly, see Equation (6). The QuickSort algorithm was used for this.

Based on these strategies, the computational complexity of the critical steps in the data processing can be estimated as a function of the frame width W, length L and number of frames T, the DoG filter length M and the user-set parameter K. The loading and data visualization module have a complexity of O(WLT), as the number of operations required to load, convert and display the image stack are directly linked to the number of pixels loaded. The separable DoG filter enables the ROI detection module to run in O(M(W+M)(L+M)) operations due to the M pixels padded at the image borders to enable ROI detection at the boundaries [30]. Last, sorting the values in a window of length K with QuickSort during the signal normalization module requires O(TKlog(K)) operations and the peak detection, which requires $L_{f}$ multiplications to calculate the SD and performs this $T-L_{f}$ times while sliding the window, needs $O(L_{f}\left(T-L_{f}\right))$ operations per ROI.

### 3.3. Processing Speed Validation

The computational complexity as well as the actual data processing speed was verified. The focus of the timing experiments was on the number of frames T for loading the data, the filter length M for the ROI detection step and the window size K for the signal extraction step. The image width and length were not varied, as the frame sizes of the footage produced by different microscopes vary less than an order of magnitude. An analysis of the frame length and width is, thus, not of interest to obtain a realistic estimate of the processing time required by CALIMA 2.0.

Subsets of the 1200 frames of *dataset 1* were loaded into the program to estimate the processing speed as a function of the number of frames T. All 1200 frames were loaded during the tests to effect of different filter lengths M on the ROI detection module. Lastly, the 51 ROIs found by using the settings on the 1200 frame stack were used as a mask to determine the effect of the sliding window length K on the operation time of the signal extraction and the window length $L_{f}$ on the peak detection module. In addition to *dataset 1*, the procedure processing the 55 frames of *dataset 2* was timed with the settings of Table 3, resulting in 3108 ROIs being detected.

All timing tests were executed on an 2016 Intel(R) Core(TM) i7-5600U CPU with 4 logical cores running Windows 7, and were repeated 5 times in order to get a fair estimate of the processing time required to complete each computational task. The execution time of each processing step was timed with C#’s StopWatch subroutine.

Loading the data, finding the ROIs and extracting the signals for with the parameter described in Table 2 took an average of 160.71 s in total for the 1200 frames, leading to a processing time of 134 milliseconds per frame for the PC used. Of these 134 milliseconds, 122.5 milliseconds were used to load the data, 3 milliseconds to find the ROIs, 8.5 milliseconds to extract the $\Delta F/F_{0}$ traces and 4.8 microseconds to detect the peaks for the 51 ROIs that were detected. Increasing M*,*K or $L_{f}$ to the most detrimental values, 600 pixels, and 1200 and 600 frames respectively, only increased the processing time per frame of the respective processes to 11.0 ms, 11.1 ms and <1 ms. This further underlines the observation that the data loading process is considered the step that takes up most processing time.

The entire data processing procedure of *dataset 2* took more time with 990 ms per frame, divided in 630.5 ms for the data loading, 87 ms for the ROI detection, 271.5 ms for the signal extraction and 1 ms for the peak detection. The reason behind this is that both the frame size and the number of ROIs detected were significantly larger for *dataset 2*.

## 4. Discussion

### 4.1. CALIMA 2.0 as a Valuable Tool for Calcium Spike Detection in BoC-Devices

Previous data regarding culturing of primary neuronal cells, hiPSCNss and SHSY-5Y cells have shown that CALIMA 1.0 is capable of determining cell connectivity and activity through CI [4,17,26]. Applying CALIMA 2.0 to the previously accessed data now showed that a physiological link can be attributed to CI-data sets with a high enough acquisition rate. Calcium influxes range from microseconds to hours, depending on their function [31]. Therefore, their amplitude and duration play a major role in the interpretation of CI-data. In neuronal calcium signaling, the calcium concentration can rise during electrical activity [32], i.e., cell signaling. Calcium transients, due to an action potential, have a fast rise and continue in a downwards slope [33]. Figure 2a presents recurring peaks in primary rat cortical cells, which can be translated to activity bursts. Two individual ROIs and their calcium transients can be seen in Figure 2d.

The hiPSNCs cultured on NG-PDMS are relatively in an early development stage (13 DIV) and demonstrate slow-varying calcium spike activity, see Figure 3. These slow calcium spikes are likely calcium waves, which can be linked to axonal extension [34]. This slow calcium activity is, thus, of great interest in the study of BoC cultures. Long-term cultures take up to months to develop, which in turn, enhances neurite outgrowth [35], thus, improving the possibility of enhanced network formation. Visual data provided by CALIMA 2.0 can easily give an overview of the differences between experimental groups. Whereas, numerical data provides the possibility for extensive, comparative data analysis.

The response of a neuronal culture to an applied stimulus can be seen in Figure 4. The results of the spatio-temporal analysis were in line with findings from a manual evaluation of the data, which was conducted in the original research [5]. The study of stimulus-based responses is particularly interesting when working towards closed-loop experiments, a future goal of BoC research.

Our results display the complexity of analyzing BoC-cultures seen in Figure 3compared to standard two-dimensional (2D)-cultures, such as those displayed in Figure 2, Figure 3 and Figure 4 also showcase the possibilities of CALIMA 2.0 in a more advanced BoC-environment due to the improvements in CI-data processing, as explained in Figure 1. Hence, while two-photon microscopy and juxtacellular electrodes is the gold standard for acquiring CI footage, CALIMA 2.0 is able to gather valuable information about a BoC culture from standard fluorescence microscope data without applying a complex image recording technique. This reduction in experimental complexity may provide an important step for the development of BoC-devices, especially in examining the relationship between the correlated activity and network activity.

Next to neuronal (BoC) research, CALIMA 2.0 can be a valuable tool for other organ-on-chip research, wherein CI-imaging is applied. For example, CI-imaging has been applied to visualize calcium signalling in skeletal muscle fibers. It is expected that the DoG ROI detection, which is based on blurring images [19], which inherently leads to fine detail reduction, and is especially well-suited for its application to objects with smooth edges, such as muscle fibers. To understand quantitative myoplasmic calcium movements, we would opt for an accurate estimate of delta calcium [36].

### 4.2. CALIMA 2.0 Operation Speed

From an analysis of the calculation speed on a medium-performance computer in a 696 by 520 pixel TIFF-format dataset studying calcium spikes, it was found that CALIMA 2.0 requires an average of 134 milliseconds of processing time per frame. A 1280 by 960 pixel data set tracking 3108 ROIs required 990 ms per frame. Hence, data that is acquired at a slower rate than 1 Hz, such as data sets studying calcium waves [4,5,25,26], could be processed directly by a theoretical closed-loop version of CALIMA 2.0, but data obtained at a fast acquisition rate of e.g., 33 Hz, which is required for calcium spike-based closed-loop experiments as were performed in [37], would have to be processed by a much faster implementation of the software.

It was found that the time required to load a frame of the footage is the bottleneck, as it takes up over 90% of the total processing time (122.5/134 ms). An online analysis construction, such as in [7] does not require the data to be saved and again loaded, and would significantly decrease the time CALIMA 2.0 would require to process a single frame. For the PC and data set used for the operation speed validation, the processing time per frame, required for the ROI detection and signal extraction would reduce to 11.5 ms, which is comparable to the maximum of 28 ms processing time required per frame by Giovannucci et al. [7]. Please note that the methodology of Giovannucci is more extensive than CALIMA 2.0’s, and includes motion artefact correction in addition to ROI detection and signal extraction. Still, the processing time of CALIMA 2.0 can be considered in the same order of magnitude as methodologies intended for closed-loop experiments. Hence, with some adjustments, CALIMA 2.0 could serve as a base for the development of closed-loop experiments, such as previously described by Mitani et al. [6], Giovannucci et al. [7] and Vogelstein et al. [37].

### 4.3. Outlook

In addition to the development of a closed-loop version, CALIMA 2.0 could be extended to facilitate the study of cellular cultures further. CALIMA 2.0 detects ROIs through discovering sharp edges in fluorescence intensity. Cells that overlap have edges that fade gradually, and CALIMA 2.0 is, thus, at risk of merging such cells into one ROI. An intensity-based declumping algorithm, as is used in Cellprofiler [38], could better differentiate between different ROIs. Furthermore, sharp edge detection makes it difficult to incorporate neurites into the ROIs, while the calcium signals in neurites can at times be observed in experiments [5], and could be a great source of information when studying network formation. In future versions, an active contours algorithm could be included to help extend the DoG-deteced edges to incorporate neurites [39]. Another issue to be solved is cell shifting. The feedback stimuli used are sometimes of a mechanical nature [5], which causes the culture to move. Hence, the ROI locations would need to be adapted after each stimulus, which could be done by correlating ROI maps.

## 5. Conclusions

CALIMA 2.0 is valuable in the analyzing CI-data in a BoC-environment. CALIMA showed to be able to identify regions of interest, translate the raw data to $\Delta F/F_{0}$ traces, detect signal peaks and correlate the activity between individual ROIs. In this work, CALIMA aided in understanding the maturity and activity of a brain-on-chip culture. To this end, CI-data was processed and essential statistics were calculated, such as the number of calcium spikes produced per region of interest or the activity correlation between regions.

Additionally, the processing speed of CALIMA was determined both theoretically and by computational tests. It was found that an average of 134 ms was required to process a single frame of CI-footage (696 by 520 pixels, dozens of ROIs), hence, taking a safety margin, CALIMA would be able to process such data faster than it is acquired for frame rates up to 5 Hz. Tracking calcium waves in thousands of ROIs is already feasible, as such, data are processed at around 1 second per frame and are typically acquired at frame rates way below 1 Hz.

## Figures and Tables

**Table 1:** The proposed start estimates for the parameters of CALIMA 2.0.

Operation	ROI Detection			Signal Extraction		Peak Detection		
**Parameter**	$\sigma_{a}$	$\sigma_{b}$	$Th_{DoG}$	K	q	$L_{f}$	$Th_{z}$	$j_{in}$
**Unit**	Pixels	Pixels	-	Frames	%	Frames	-	-
**Value**	3.0 to 10.0	$1.6\sigma_{a}$	$0.002\sigma_{b}/\sigma_{a}$	>calcium spike duration	10–20	>calcium spike duration	≥3.0	0.1–0.2

**Table 2:** The parameters used to test the processing speed of CALIMA 2.0 and detect physiological levels of calcium influx in primary rat cortical neurons in *dataset 1*.

Operation	ROI Detection			Signal Extraction		Peak Detection		
**Parameter**	$\sigma_{a}$	$\sigma_{b}$	$Th_{DoG}$	K	q	$L_{f}$	$Th_{z}$	$j_{in}$
**Unit**	Pixels	Pixels	-	Frames	%	Frames	-	-
**Value**	6.6	10.6	0.0030	25	10	10	5.0	0.2

**Table 3:** The parameters used to visualize early calcium activity of hiPSCNs in dataset 2.

Operation	ROI Detection			Signal Extraction		Peak Detection		
**Parameter**	$\sigma_{a}$	$\sigma_{b}$	$Th_{DoG}$	K	q	$L_{f}$	$Th_{z}$	$j_{in}$
**Unit**	Pixels	Pixels	-	Frames	%	Frames	-	-
**Value**	1.0	2.5	0.0040	10	30	8	5.0	0.2

**Table 4:** The parameters used to visualize spatio-temporal activity in dataset 3.

Operation	Signal Extraction		Peak Detection		
**Parameter**	K	q	$L_{f}$	$Th_{z}$	$j_{in}$
**Unit**	Frames	%	Frames	-	-
**Value**	10	30	20	5.0	0.5

## Data Availability

The hiPSNCs data presented in this study and CI-tool CALIMA 2.0 are openly available on GitHub at https://github.com/EALRaaijmakers/CALIMA-2.0. A publicly available dataset of primary rat cortical neurons was analyzed in this study. This data can be found here: https://www.seas.upenn.edu/~molneuro/software.html, [22]. The CI-data related to the response evoked by an external stimulus was obtained from the supplementary material of [5].
